# Michael Marmot: the health of nations

**DOI:** 10.1192/bjb.2022.81

**Published:** 2023-02

**Authors:** Claire McKenna

**Figure:**
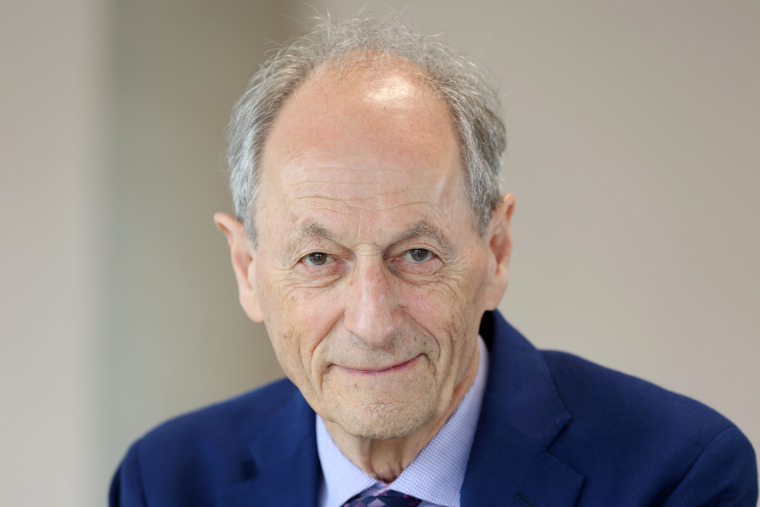

‘I'm worried it will be a humanitarian calamity in the fifth richest country in the world.’

Michael Marmot on the unprecedented challenge of the UK's cost of living crisis.

‘My difficulty saying no is haunting me,’ says Professor Sir Michael Marmot, when I talked to him via Zoom in July 2022. He has squeezed in this interview at the end of a day that included: making a podcast on leadership with a young GP; various meetings in his role as Director of the UCL Institute of Health Equity; and giving a lecture. ‘Last year I looked at the folder on my computer where I keep presentations and I counted it up. Last year I gave 143 PowerPoint presentations and there were some lectures I did without PowerPoint. So I was giving a lecture twice in three days on average for the whole year.’

The 77-year-old's output in print and media is prolific. The central thesis of his life's work – that ‘social injustice is killing on a grand scale’ – is now familiar to many, in no small part because he has made it so. Marmot began his 2015 book *The Health Gap* with the line: ‘What good does it do to treat people and send them back to the conditions that made them sick?’^1^ This driving force has animated his academic and campaigning work since his time as a young doctor in Australia, his country of birth.

As past President of the World Medical Association and through many reports (most notable in the UK is his eponymous 2010 review of health inequalities in England^2^), Marmot has made an impassioned but evidence-based case for improving health outcomes by reducing inequality. ‘Most of the things that doctors treat are failed prevention,’ he has said.

The UK government's ‘levelling up’ agenda suggests his ideas have reached the mainstream, although at the time of writing, the spectre of the 2022 White Paper^3^ being scrapped looms large. ‘The levelling up White Paper should be entirely consistent with my ideas and the levelling up commitment. But you've got to put real effort into it,’ he cautions. ‘You can't just say “We're for levelling up”. And when it comes to economic growth [in reducing health inequalities], it depends on what kind of growth. Is it inclusive? Economic growth that just makes rich people richer isn't going to do much for health inequalities. “Trickle down”’ – forget it – it doesn't work. That's not a viable economic model.’

Marmot linked austerity to the first fall in life expectancy for 100 years, in his updated report on health inequalities in England in 2020.^4^ There's something of a ‘last days of Rome’ feel in the air at the moment, with even the Bank of England governor using apocalyptic language^5^ to describe the economic tundra rolling out before us. Marmot has issued a political call to arms on the cost of living crisis, in several *Guardian* opinion pieces.^6,7^

For psychiatrists, the call could not be more relevant. He says the evidence points to the mind as the mechanism by which structural inequalities mediate poor health. A recurrent theme of Marmot's is the sheer intellectual energy it takes to be poor. In Glasgow, for example, he points out that the biggest causes of excess deaths (suicide, alcohol and drugs) ‘are all psychosocial in origin – they are what happens when people are disempowered, and have little control over their lives’.

Does he feel a tug of duty to engage the government and public on the social determinants of health? He admits to some sense of duty, but insists that his drive for social justice emerges from the evidence, rather than ideology. He has tried to steer away from party politics.

Amidst the catastrophic language he is surprisingly upbeat and answers my questions with brio. ‘We're on the winning team,’ he maintains. ‘I'm trying to get people engaged with the whole idea that the principle of social justice in health can be made real by action on the social determinants of health. And the more people I get signed up to that, the better.’

Forty minutes into our conversation, Zoom impertinently announces that I need to upgrade in order to continue. I have lots more questions for Professor Marmot, but I decide not to ask any more of him. He looks like he's had a long day. I remember the 143 PowerPoint presentations a year. Despite his claim that he has only doggedly followed the science, it seems like a vocation of one sort or another to me.

The following interview has been edited for length and clarity.

**How would you answer people who say ‘Of course you can live on 50 p a day. The poor don't know how to budget and they should learn to cook’?**

I'd say, try it. Try it. And if you don't want to try it, read Jack Monroe's blog ‘Cooking on a Bootstrap’ (https://cookingonabootstrap.com/category/blog). Here's a resourceful person who knows how to cook and also knows what it's like to suffer the depression of leading a life with no dignity because of low income. We published data from the Food Foundation that shows if you're in the bottom 10% of household income, to follow the healthy eating advice, you would have to spend 74% of your income on food.

The evidence is that poor people know how to cook as well as richer people. It's not lack of knowledge. It may be depression. It may be lack of money. They can't afford the food.

But again, read Jack Monroe's blog. If you or I go to the supermarket, we might say ‘Gosh, carrots are a bit more expensive’ or ‘Do you know what it's like to buy dried figs now?’ The price has gone up, but we pop them in the trolley and we check out. But if you're on a tight budget, you go round with a pad of paper and check all the prices. And then you add it up: ‘Nope. I'm £1 over. I don't have that extra pound to spend. I better go back round again’. It takes much longer. It takes a huge amount of intellectual energy to try and fit within the budget. You don't say ‘The price of carrots and dried figs has gone up’. You say ‘Who can afford dried figs? That's a luxury beyond me and I may have to do without fresh carrots [ … ] Dry pasta and a can of chopped tomatoes will have to do it for tonight, thank you’.

And then some comfortable, rich person says ‘I could live on that amount of money’. No. Do me a favour.

**Your career has focused on using data and statistics to highlight health inequalities, but your strong moral compass and your belief in human dignity really comes across in all your work as well. And that's not based on the evidence. Where do you get your strong sense of social justice from?**

Well, in a sense, it does come from the evidence. Firstly, if you take the idea of dignity: when I started doing research on social inequalities in health, I was committed to the idea that psychosocial influences were important; that the mind is not the only, but an important, gateway by which social circumstances have an impact on health. Dignity means the quality of people's lives and their relationships, how much agency they have and how much control over their lives. We found that these were all important to health. And that was a fundamental approach supported by evidence.

When we were doing the Commission on Social Determinants of Health in the Americas, one of our commissioners from Bolivia talked very much about dignified lives. And that resonated with me. I had talked until then about ‘empowerment’. But part of empowerment, having agency, is leading a life you have reason to value. That's an Amartya Sen [Indian economist and philosopher] concept. Leading a life you have reason to value. For most people, a life of dignity is a life they have reason to value.

And that's why I placed great emphasis on that in that opinion piece the *Guardian* asked me to write, about the cost of living crisis and health.^6^ And the reason I put dignity at the centre of it is because of this interest in psychosocial processes. You said that the commitment to social justice didn't come from the data; when I started to do research on social inequalities in health, it very much did come from the data. And as I went on, I got more and more exercised as I believed that what the data was showing me was if we change things, we could make a difference.

I chaired the World Health Organization Commission on Social Determinants of Health^8^ (2005–2008), and it was my phrase I put in the submitted report: ‘social injustice is killing people on a grand scale’. Because I think the evidence showed that we had remediable causes of health inequalities. And as a prime example I quoted at the time: we said that for the 1 billion people in the world who were living in slums, it would cost $100 billion to upgrade those slums. This was back in 2008; the figures would be slightly different now. And then I thought ‘Who's going to find $100 billion for anything? That looks ridiculous’. You let academics loose on a topic and they come up with things like ‘We need $100 billion to upgrade the slums’.

Then we had the global financial crisis. And we found $11 trillion to bail out the banks. And I said, ‘For 1/110th of the money we found to bail out the banks, every urban dweller in the world could have clean running water'. And the fact that they don't is a matter of deep social injustice because we could do it. Technically we could do it. We know what to do. We have the means to do it, and if we're not doing it, that's wrong. That's social injustice.

**Why do you call the cost of living crisis in the UK ‘unprecedented’?**

I quoted in that *Guardian* piece^6^ figures from The Resolution Foundation [illustrating the severe impact the crisis will have on household incomes]. We haven't seen something on that scale for a very long time. The average person, they will be 4% worse off. In general, if you're a single person out of work, your income will go down by 15%. Wow. That's why I called it unprecedented.

We think there's more than income that matters for health, but if you're at the margin and you lose income, then it matters enormously.

I talk about absolute versus relative poverty. I've been wrestling with this question all my research life and policy life. Are they both important? Yes, they're both important. If you're below the minimum income threshold, if your income is less than 60% median income, absolute poverty really matters.

But relative poverty matters too. What does relative poverty mean? I quote the Adam Smith idea of taking your place in public without shame. If all the other kids on the street have a new pair of trainers or the latest football kit, if all the other kids on the street have a birthday party when it's their birthday, but you don't, you can't take your place in public without shame. And as a parent, you're condemning your child to shame and that's shameful on you and a loss of dignity. So it's a psychosocial concept.

And the final influence that determines how much loss of income will matter to people is about this discussion between universal basic income and universal basic services. If you think about basic services – education, healthcare, housing, food, transport, communication – the cheaper they are, the less individual income matters. Now, communication – broadband access is a basic need, isn't it? And it wasn't a basic need 20 years ago, but it is now a basic need. You can't participate in society if you've got no access to broadband. And if it's very expensive, you're priced out.

So there's an argument going on. There are those who are committed to universal basic income and others who are committed to universal basic services. I think the answer is somewhere in between. You need enough money, no question. But the better the services, the less money you need.

**What are you worried about if this cost of living crisis continues unchecked?**

I'm worried that the impact on the lives people are able to lead will be unprecedented and that it will be a humanitarian calamity in the fifth richest country in the world.

I've said we should put equity of health and well-being at the heart of all government policy. My emphasis is on the social determinants of health, not on the healthcare system. But by goodness, the mess they've made of the healthcare system. Ten years of underfunding: boasting that they ring-fenced the NHS and yet systematically underfunding it.

Yes, there's the pandemic. But we were so ill prepared for it. How many hospital beds did we lose over the last decade? So we're short of doctors, short of nurses, short of hospital beds because of years of underfunding. It's just not our priority to fund public services as a government.

I heard the health secretary on the radio saying ‘We're going to build 48 new hospitals by 2030’, as if somehow that will solve a decade of underfunding and demoralisation. What about the decade of destruction that got us to this terrible position?

**You've talked about the importance of doctors being able to shift our gaze from firefighting – because it does feel like firefighting in the NHS at the moment. You have said that your perspective on what doctors could do shifted when you were President of the World Medical Association. Can you tell us a little bit about your advice for doctors? What can we do to address health inequalities?**

Well, when I started to represent the British Medical Association on the Council of the World Medical Association, I thought ‘What on earth am I doing here?’ These are medical associations and as most medical associations do, they're concerned with pay and conditions of doctors and important things like ethics and medical research and so on. But I thought, still, ‘That's not me’. And then when it was suggested that I might put my name forward to become President, I thought ‘I think you've got the wrong person, not me’. And they said ‘No, we'll support you’.

So I made it very clear I wanted doctors to get engaged on the social determinants of health, and they were very receptive. We developed a report on what doctors could do^9^ and we noted five things.

First, education and training on social determinants of health.

Second, seeing the patient in broader perspective. You don't treat a rough sleeper and send them back onto the street to sleep rough, or at least you shouldn't. You shouldn't stitch up a woman's cuts and bruises and send her back to an abusive partner.

Third is what I would now call ‘the health system as anchor institution’. But at the time, I said ‘the health service as employer’. Good conditions for employees, not just doctors and nurses, but drivers, lab technicians, secretaries and the like.

Fourth, working in partnership with people.

And fifth, advocacy. And in a way, that's part of what I've been doing. Giving 143 PowerPoint presentations last year was advocacy!

**Do you think doctors need to be activists?**

I'm not good at telling people what they should be doing. People should do what they feel is the right thing to do. I'm quite good at telling governments what they should be doing, but not colleagues.

But there's a clear role for doctors to be advocates on behalf of the patients that they serve and the communities that they serve. And our profession is highly trusted by the population. Surveys show that. Ask people ‘Who do you trust to tell the truth?’ Regularly, number one: nurses. Number two: doctors. We're right up there just behind our nurse colleagues. It's quite nice that nurses are the most trusted. Why would you not trust nurses? Best people in the world. But we're not far behind.

That trust has been earned. We must be careful not to betray that trust, but use the position of trust to speak up on behalf of the health of the patients and populations that we serve. So, yes, I wouldn't say you *should* or you *need* to become an advocate, but there's a clear role for you to play, if you're prepared to do it.

## References

[1] Marmot M. The Health Gap: The Challenge of an Unequal World. Bloomsbury, 2015. 10.1016/S0140-6736(15)00150-6 26364261

[2] Marmot M, Allen J, Goldblatt P, Boyce T, McNeish D, Grady M Fair Society, Healthy Lives: The Marmot Review. Strategic Review of Health Inequalities in England Post-2010. The Marmot Review, 2010 (https://www.parliament.uk/globalassets/documents/fair-society-healthy-lives-full-report.pdf).

[3] HM Government. Levelling Up: Levelling Up the United Kingdom (CP604). HMSO, 2022.

[4] Marmot M, Allen J, Boyce T, Goldblatt P, Morrison J. Health Equity in England: The Marmot Review 10 Years On. Institute of Health Equity, 2020 (health.org.uk/publications/reports/the-marmot-review-10-years-on). 10.1136/bmj.m693 32094110

[5] Bailey A. Treasury Committee: Oral evidence: Bank of England Monetary Policy Reports, HC 143 (16 May 2022): Q437. House of Commons, 2022 (https://committees.parliament.uk/oralevidence/10215/pdf/).

[6] Marmot M. Studying health inequalities has been my life's work. What's about to happen in the UK is unprecedented. Guardian 2022: 8 Apr (https://www.theguardian.com/commentisfree/2022/apr/08/health-inequalities-uk-poverty-life-death).

[7] Marmot M. A generation of Britons face long-term illness from being cold and poor this winter. Guardian 2022: 1 Sep (https://www.theguardian.com/commentisfree/2022/sep/01/generation-britain-long-term-illness-cold-poor-winter-cost-of-living-crisis).

[8] World Health Organization. Commission on Social Determinants of Health. Closing the Gap in a Generation: Health Equity through Action on the Social Determinants of Health: Commission on Social Determinants of Health Final Report. World Health Organization, 2008.

[9] Thomas S. Doctors for Health Equity. TSO (The Stationery Office), 2016 (https://www.instituteofhealthequity.org/resources-reports/doctors-for-health-equity-world-medical-association-report/doctors-for-health-equity-wma-full-report-pdf.pdf).

